# Needle fasciotomy versus limited fasciectomy for the treatment of Dupuytren’s contractures of the fingers (Hand-2): study protocol for a randomised controlled trial

**DOI:** 10.1186/s13063-024-08003-1

**Published:** 2024-06-19

**Authors:** Samantha Harrison, Reuben Ogollah, William Hollingworth, Nicola Mills, Alexia Karantana, Jane Blazeby, Alan Montgomery, Aisha Shafayat, Hugh Jarrett, Tim Davis

**Affiliations:** 1 Nottingham Clinical Trials Unit, University of Nottingham, Building 42 Applied Health Research, Nottingham, NG7 2 UK; 2 Bristol Medical School, Population Health Sciences, University of Bristol, Bristol, BS8 2PS UK; 3 Bristol and Weston Biomedical Research Centre, University Hospitals Bristol NHS Foundation Trust and University of Bristol, Bristol, UK; 4 Queen’s Medical Centre, Nottingham University Hospitals NHS Trust, Derby Road, Nottingham, NG7 2UH UK

**Keywords:** Dupuytren’s contractures, Needle fasciotomy, Limited fasciectomy, Randomised control trial, Patient acceptability, Hand surgery

## Abstract

**Background:**

Dupuytren’s contractures (DC) are fibrous cords under the skin of the hand that cause one or more fingers to curl gradually and irreversibly towards the palm. These contractures are usually painless but can cause a loss of hand function. Two treatments for Dupuytren’s contractures are widely used within the National Health Service (NHS) in the UK: removal of the contractures via surgery (limited fasciectomy) and division of the contractures via a needle inserted through the skin (needle fasciotomy).

This study aims to establish the clinical and cost-effectiveness of needle fasciotomy (NF) versus limited fasciectomy (LF) for the treatment of DC in the NHS, in terms of patient-reported hand function and resource utilisation.

**Methods/design:**

Hand-2 is a national multi-centre, two-arm, parallel-group randomised, non-inferiority trial. Patients will be eligible to join the trial if they are aged 18 years or older, have at least one previously untreated finger with a well-defined Dupuytren’s contracture of 30° or greater that causes functional problems and is suitable for treatment with either LF or NF. Patients with a contracture of the distal interphalangeal joint only are ineligible. Eligible consenting patients will be randomised 1:1 to receive either NF or LF and will be followed up for 24 months post-treatment. A QuinteT Recruitment Intervention will be used to optimise recruitment. The primary outcome measure is the participant-reported assessment of hand function, assessed by the Hand Health Profile of the Patient Evaluation Measure (PEM) questionnaire at 12 months post-treatment. Secondary outcomes include other patient-reported measures, loss of finger movement, and cost-effectiveness, reported over the 24-month post-treatment. Embedded qualitative research will explore patient experiences and acceptability of treatment at 2 years post-surgery.

**Discussion:**

This study will determine whether treatment with needle fasciotomy is non-inferior to limited fasciectomy in terms of patient-reported hand function at 12 months post-treatment.

**Trial registration:**

International Standard Registered Clinical/soCial sTudy ISRCTN12525655. Registered on 18th September 2020.

**Supplementary Information:**

The online version contains supplementary material available at 10.1186/s13063-024-08003-1.

## Introduction

Dupuytren’s contractures (DC) are fibrous cords under the skin of the palm of the hand. They typically occur in men and women over 50. They have a strong genetic tendency and increased incidence associated with diabetes and epilepsy [1]. The contractures are painless but cause one or more fingers to gradually and irreversibly curl into the palm, resulting in loss of hand function [2, 3]. The standard treatment is surgery to remove or divide the Dupuytren’s contractures, allowing the finger to straighten (extend) again. Surgery, however, does not cure Dupuytren’s contractures, and recurrent contractures may occur and require further treatment [4].

Two surgical treatments for troublesome Dupuytren’s contractures are commonly undertaken. One is “limited fasciectomy” (LF), in which the fibrous cords preventing the finger(s) from straightening are cut out of the hand through a long skin incision. This procedure is typically done under general or regional anaesthesia in an operating theatre and has a 4–6-week recovery period. The other surgical treatment is “needle fasciotomy” (NF), in which the fibrous cords preventing the finger(s) from straightening are divided with the sharp tip of a hypodermic needle which is passed through the skin into the underlying fibrous cord. NF can be done in an outpatient clinic room and has a 1- to 2-week recovery period.

Initially NF is less expensive for the NHS, less disruptive for patients, and probably carries a lower risk of complications that restrict hand function [5]. However, the risk of a recurrent contracture forming and bending up the finger again is greater after NF than after LF [6]. Recurrence may necessitate further treatment and increase costs. Also, LF may straighten the finger better than NF.

Systematic reviews of the surgical treatment for DC [7–13] have shown that there is no high-quality research demonstrating whether NF or LF is superior to the other in terms of preserving hand function and “value for money” to the health provider. The lack of well-designed and conducted trials means that the choice of treatment for Dupuytren’s contractures of the fingers mainly depends on surgeon and patient preference [14]. NHS England produced guidance on intervention for Dupuytren’s contracture in 2018 (published 28.11.18 [15]) which stated “No-one knows which interventions are best for restoring and maintaining hand function throughout the rest of the patient’s life, and which are the cheapest and most cost-effective in the long term.” Also, the James Lind Alliance Priority Setting Partnership for Hand Surgery [16] found the research question: “In patients with Dupuytren’s disease, what techniques give the best results in terms of function, recurrence and cost?” was a top 10 priority.

This study aims to answer some of the many uncertainties regarding the optimum treatment of Dupuytren’s contractures. The RCT comparing NF and LF will examine their relative values in terms of clinical outcome, costs and acceptability to patients over a 2-year follow-up period. Also, this study has been designed to allow an indirect comparison of the outcomes of another treatment for Dupuytren’s contractures, collagenase (which was studied in another trial (DISC; ISRCTN18254597) [17]), with NF treatment in a network meta-analysis which will use individual patient data. This is not described in this protocol paper.

### Objectives

The objectives of the Hand-2 study are:

1. To determine whether, in adults with symptomatic DC of the hand, treatment with NF is non-inferior to LF in terms of hand function (assessed with the Hand Health Profile of the PEM) at 12 months post-treatment.
2. To compare NF and LF with respect to:
  Participant-reported hand function and overall satisfaction at 3 weeks, 6 weeks, 3 months, 6 months and 24 months using the Hand Health Profile PEM questionnaire.
  Participant-reported assessment of location-specific health (the hand) using the Single Assessment Numeric Evaluation (SANE) tool at 2 weeks, 3 weeks, 4 weeks, 6 weeks, 3 months, 6 months, 12 months and 24 months and the Measure Yourself Medical Outcome Profile (MYMOP) tool at 3 weeks, 6 weeks, 3 months, 6 months and 24 months.
  Loss of finger extension at 6 weeks and 6, 12 and 24 months
  Adverse events and complications, recurrence of DC, and revisions or salvage surgery up to 24 months
  General health-related quality of life using the EuroQol (EQ-5D-5L) questionnaire, resource use, and cost-effectiveness at 2 weeks, 3 weeks 6 weeks, 6 months, 12 and 24 months
  Treatment acceptability at 2 years post-surgery (integrated qualitative research)

## Methods

This protocol follows SPIRIT reporting guidelines [18, 19].

### Trial design

Hand-2 is a multi-centre, two-arm, parallel-group, randomised, non-inferiority trial comparing the outcome of NF and LF among adults eligible for treatment of DC within the NHS. Participants will be allocated on a 1:1 ratio to treatment with either LF or NF and followed up for 24 months post-treatment. A QuinteT Recruitment Intervention (QRI) [20] will be embedded within the trial to enable optimisation of the recruitment phase.

**Fig. 1:**
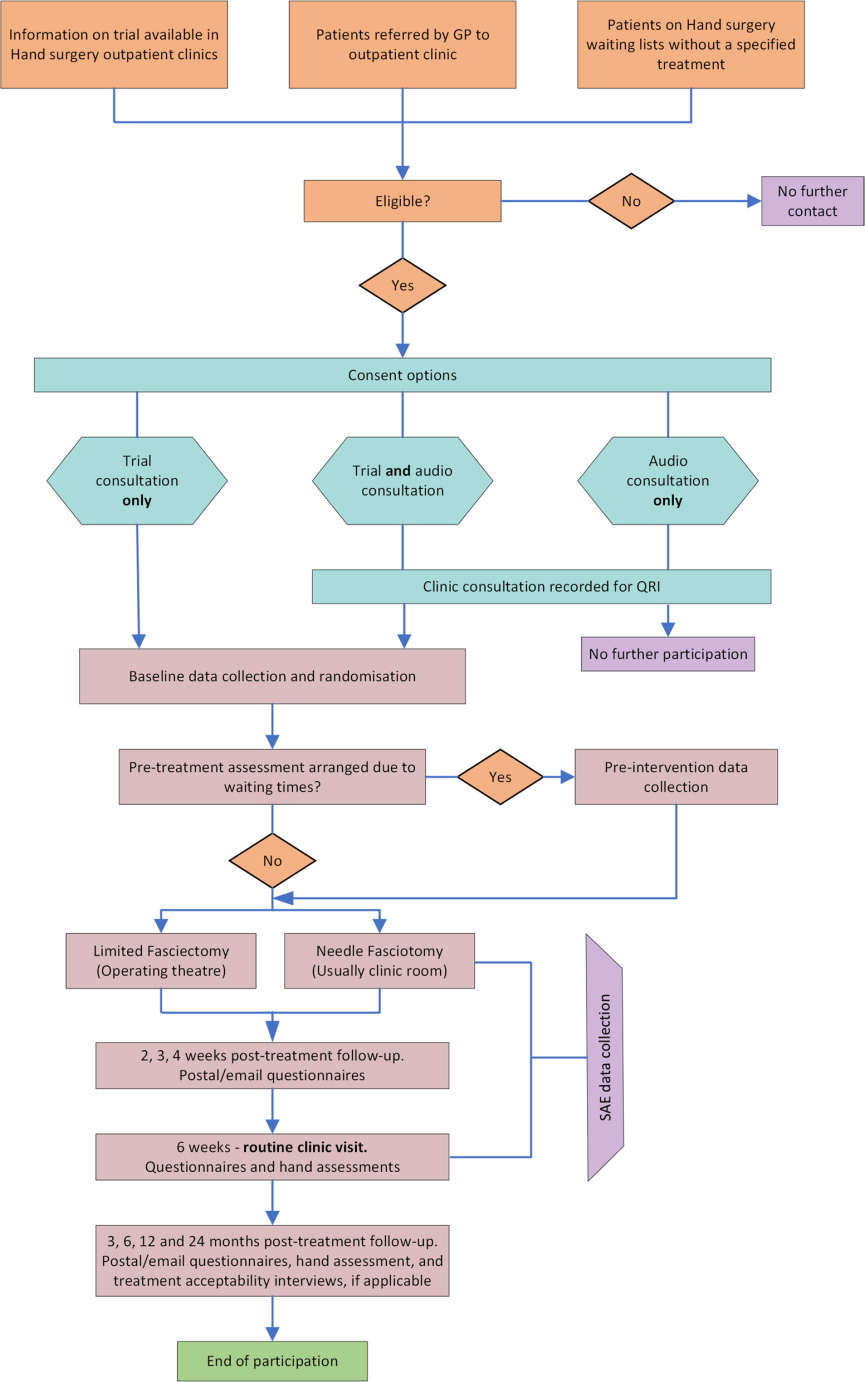
Participant pathway

The participant pathway is outlined in Fig. 1.

### Trial setting

Patients will be recruited from and treated at UK secondary care centres. A list of participating centres can be found at https://www.isrctn.com/ISRCTN12525655.

### Participants and recruitment

Twenty-one secondary care sites in England and Scotland will conduct screening and recruitment of participants for the Hand-2 study.

#### Eligibility criteria

Eligibility criteria are outlined in Table 1.

**Table 1:** Eligibility criteria for the Hand-2 study

Inclusion criteria	Exclusion criteria
Aged 18 years or older	Dupuytren’s contracture of the distal interphalangeal joints (DIP) only
One or more fingers with a Dupuytren’s contracture of 30° or greater with functional problems	Planned dermofasciectomy or very limited fasciectomy (excision of ≤ 1 cm cord segment)
No previous treatment for Dupuytren’s contracture on study finger	Previously recruited into the Hand-2 study for treatment of either hand
Well defined cord(s) Suitable for treatment with either NF or LF	
Able to comply with the requirements of the study up to 24 months post-treatment	

#### Recruitment

Recruitment will take place from March 2022 to March 2024. Participants will be recruited from 21 NHS sites across England and Scotland. Two recruitment pathways will be used to maximise recruitment:

1. The main recruitment pathway will be via secondary care elective outpatient hand clinics. Potential participants will be identified before their NHS clinic appointment by screening of GP referral letters and clinic lists by the local clinical care and/ or local research team at sites. A short patient information leaflet will be sent to potentially eligible patients explaining Dupuytren’s contractures and the study before their clinic appointment. The leaflet will also explain that if they are potentially suitable for the study they may be asked for permission to audio-record consultations with the surgeon and local research team during the clinic visit. Audio recordings will form part of the Quintet Recruitment Intervention (QRI; outlined below), which aims to optimise the recruitment process.
2. An alternative recruitment pathway will identify potentially eligible participants who have been placed on a surgical waiting list for “surgery for Dupuytren’s contracture” and not for a specific procedure such as LF or NF. Potential recruits will be sent a short Patient Information Leaflet at least 4 weeks before the intervention date. They will then be contacted/seen at least 2 weeks before the day of surgery for a discussion of the trial by someone trained in trial recruitment and competent to explain the benefits and drawbacks of both limited fasciectomy and needle fasciotomy. Those willing to participate in Hand-2 will be seen face to face before the day of surgery to confirm suitability for either procedure and their willingness to participate in the trial, obtain consent and collect baseline data.

##### QuinteT Recruitment Intervention (QRI)

Recruitment and informed consent will be optimised by an embedded QRI — a flexible, tailored intervention to identify and address recruitment difficulties as they arise in study sites [20]*.* The QRI has been applied to over 60 RCTs to date, including Hand-1 [21] leading to insights about recruitment issues and the development of targeted strategies that can improve recruitment rates [22, 23].

Interviews with TMG members and trial recruiters will investigate their perspectives on the RCT and experiences of recruitment. Detailed eligibility and recruitment pathways will be compiled for clinical sites. These recruitment pathways will be compared with details specified in the trial protocol and pathways from other sites to ensure practices support efficient recruitment. Screening logs of potential RCT participants will be scrutinised, to help identify points at which they do not continue with recruitment to Hand-2 and reasons for this.

Clinic appointments in which the study is discussed will be audio-recorded, with consent, to explore study information provision, recruitment techniques, patient concerns, and randomisation decisions to identify recruitment difficulties and improve information provision. These audio recordings will be reviewed by the QRI lead, NM, and personalised one-to-one feedback will be given to recruiters where specific difficulties or sensitive issues may need to be discussed. Additionally, group feedback will be given, supported by the use of anonymised quotes, to highlight commonly identified issues and good practice. The QRI will continue throughout the recruitment period with close monitoring of changes in screening log data and recruiter practice to optimise recruitment and informed consent.

### Consent

Potential participants will be provided with participant information sheets and will discuss the study with their surgeon before giving consent to take part. Written informed consent for each participant will be obtained prior to performing any trial-related procedure. The potential participant will be given the opportunity to ask questions throughout the process. Patients will be informed that they will be free to withdraw consent to participate at any time; however, all data collected up to the point of withdrawal will be retained and used in the analysis. The 24-month follow-up will capture early DC recurrences, but it is anticipated that, subject to funding and necessary approvals, this will be extended to 5 years and so participants will be asked to consent to this longer follow-up during recruitment.

#### QRI and integrated qualitative research consent

Patients will complete separate written consent for consultation audio-recordings for use in the QRI. Patients will be able to consent to the audio recordings without consenting to the main trial, and vice versa. Written consent for consultation audio-recording and research interview will also be sought from site and study staff as part of the QRI. Separate written consent will be obtained for those who agree to a patient interview as part of the integrated qualitative research component.

### Randomisation and blinding

Eligible patients who consent to participate will be individually allocated on the day of recruitment on a 1:1 ratio, minimised by treating centre, hand dominance, number of fingers to undergo treatment (one or more than one) and finger joint involvement and retaining a random element, to have their DC treated by either NF or LF. If a patient presents with two or more fingers on the same hand requiring treatment, then both/all fingers will receive the same treatment (i.e. all with LF or all with NF). At recruitment, but before randomisation, the patient will be asked which finger causes them the most trouble. This will be deemed the study finger for any outcomes requiring reference to a single finger. Participants will be informed of their intervention allocation on the day of randomisation by site staff and will be placed on the NHS waiting list for their allocated treatment. Allocation will be concealed using a web-based minimisation algorithm developed and maintained by the Nottingham Clinical Trials Unit and held on a secure server, accessed by appropriately trained site staff via a secure website.

Blinding of treating surgeons and participants is not possible for this trial as the treatments are very different. Blinding of research staff will not be feasible due to the differing nature of the two surgery procedures and their recovery. Further, any attempt to cover scars in the affected hand (i.e. via latex gloves) can impact on the clinical outcome measurements performed. The trial statisticians will remain blind to treatment allocation until after database lock. An independent, unblinded statistician will generate closed reports for the Data Monitoring Committee.

### Interventions

#### Limited fasciectomy

LFs will be performed in an operating theatre or minor operating room, usually under general or regional anaesthetic, using the surgeon’s favoured skin incision. For contractures involving the metacarpophalangeal (MCP) joint, the cord will be excised proximally at least to the proximal margin of the transverse fibres of the palmar aponeurosis. Digital cords will be excised completely from their origin. In all cases, the distal margin of the cord excision will be the insertion of the cord onto the flexor sheath (or other structure). The planned fasciectomy must be a LF with the excision of much of the length of the cord, and not a very limited fasciectomy (removal of only a small length (~ 1 cm) of the cord) or dermofasciectomy (replacement of the skin overlying the cord with a skin graft).

#### Needle fasciotomy

NFs will be performed in a clinic room or operating theatre under local anaesthetic. A standard 19G–23G hypodermic needle on a syringe will be used. The DC cord will be divided at one or more levels by either: performing side-to-side movement of the needle tip across the cord, or: multiple needle punctures, in order to prevent the cord from tethering the finger.

All surgical procedures will be carried out by either a consultant surgeon, an experienced trainee, or an inexperienced trainee under direct supervision of their trainer. The surgeon’s level of experience will be recorded, along with elements of the procedure such as surgical findings and complications, procedure timings, clinical staff present during the procedure, and equipment used.

Rehabilitation after both interventions will be according to local practice and individual patient needs. It may, or may not, include supervised therapy, a formal instruction sheet and/or night splints.

### Outcomes

Outcomes will be collected at baseline (during recruitment but before randomisation) and at specific times over 24 months following treatment. Time zero for follow-up for this study will be the time of the intervention not the time of randomisation. Post-treatment, rather than post-randomisation, time points are necessary as treatment cannot be provided immediately due to NHS waiting lists. Follow-up schedules are the same across both treatment arms.

**Fig. 2:**
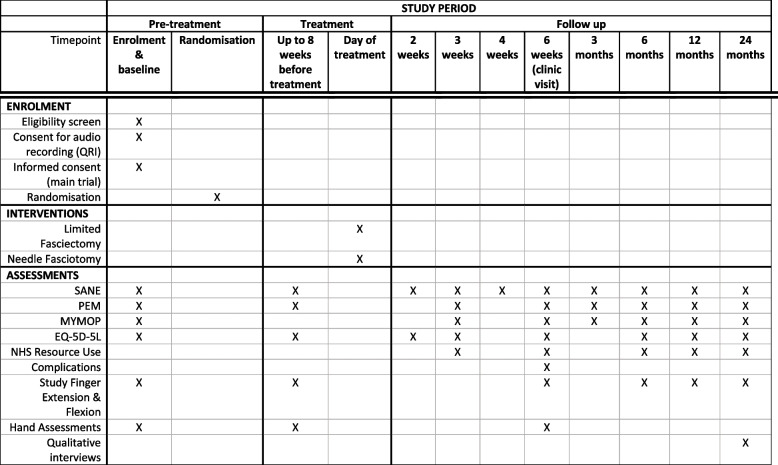
SPIRIT figure — schedule of enrolment, interventions and assessments

Post-treatment follow-ups will be completed at 2, 3, 4 and 6 weeks and at 3, 6, 12 and 24 months for participants in both study arms. Selected outcome measures will also be collected during the 8 weeks before the intervention, sometimes on the day of the intervention. This is to determine whether there has been a change (i.e. progression of contracture or development of a comorbidity impacting on hand function) between the baseline and the intervention. Please see Fig. 2 for the assessment schedule.

#### Patient-reported outcome measures (PROMs)

A range of patient-reported outcome measures will be used to assess hand function, quality of life and overall satisfaction at baseline, pre-intervention, and post-treatment:

- Hand Health Profile of the PEM [24]. This will be the primary outcome measure when collected at the 12-month follow-up.
- Single Assessment Numeric Evaluation (SANE) [25]
- Measure Yourself Medical Outcome Profile (MyMOP) [26]
- EuroQol-5 Dimension 5 level questionnaire (EQ-5D-5L) [27]

Participants will also be asked to report hand-related NHS care (e.g. splint use; primary care; medications; outpatient; hospital readmissions), social care (e.g. help with usual activities), private treatment, and employment using a resource use questionnaire.

#### Clinical outcome measures

The following objective hand assessments will be conducted at baseline, pre-intervention and post-treatment:

- Extension and flexion in the study finger
- Grip strength in both hands
- Active and passive study finger joint measurement of the study finger
- Two–point discrimination sensation at the tip of the study finger

Additionally, adverse effects of treatment (complications) will be collected post-treatment.

### Sample size

It was not possible to ascertain an estimate of the minimum clinically important difference from the Hand-1 pilot data due to the small number of participants who felt “a little better” after treatment [28]. However, the DISC trial team estimated a 6-point difference on the PEM at 1 year to represent the threshold at which treatment difference becomes important in this patient population, and which would represent an appropriate non-inferiority margin. Therefore, Hand-2 will use the six-point difference in the PEM at 12 months as the non-inferiority margin. This is equivalent to approximately a one-point reduction in the scores of six of the 11 questions in the PEM (score range for each = 1–7). From the Hand-1 pilot study, a standard deviation in the PEM of 15.1 at 6 months follow-up was observed. To avoid possible underpowering from potential imprecision in the variance estimate from pilot data, the upper 80% confidence limit of 16.6 was used.

The sample size required to achieve 90% power to detect non-inferiority of NF compared to LF within a margin of 6.0 on the PEM at 12 months using SD of 16.6 (a standardised effect size of 0.36), based on 2.5% 1-sided alpha is 324 (162 per arm). Allowing for up to 20% loss to follow-up at 12 months, the target sample size is 406. From the pilot study, an 85% follow-up of participants at the 6-month follow-up appointment was achieved without recourse to financial or other incentives. Surgeon effect has not been adjusted for as it is expected that there will be multiple treating surgeons at each site (≥ 3), with each surgeon treating a small number (≤ 7) of study participants, and at least half of the surgeons able to perform both procedures. Any treatment-related clustering is therefore assumed to be ignorable. Efforts will be made to maximise adherence with allocated treatment and incentives will be given to maximise follow-up.

### Follow-up

After randomisation, participants will be placed on the NHS waiting list for their allocated treatment. Participants will be followed up for 24 months after they receive their treatment. See Fig. 2 for timepoints and outcome measures collected.

All follow-ups will be conducted via questionnaires sent to participants’ homes via post or email, with the exception of the 6-week follow-up timepoint which will be conducted in the clinic at the patient’s standard post-operative appointment. Participants may be invited to take part in qualitative interviews up to 2 years after treatment as part of the integrated qualitative research component.

Retention will be monitored at all timepoints throughout the trial. Our primary method of patient retention is to send participants a high street voucher (of modest value) as a token of appreciation for completion and return of the questionnaires at 12 and 24 months. Data arising from the QRI and participant interviews that address issues relating to retention will be reviewed and actions implemented based on this.

#### Internal pilot phase and progression criteria

Recruitment and retention will be continuously monitored throughout the trial. A formal review of recruitment will occur 10 months after the randomisation of the first participant and will be measured against the overall recruitment target (see Table 2 for progression and stopping criteria). Due to the initial staggered recruitment, little data will be available on retention and, particularly, the 12-month primary outcome. Therefore, a further formal review of retention will occur 18 months after the first person is randomised. Retention will be assessed as the proportion of participants attending 6-week and 12-month follow-up visits (see Table 2 for progression and stopping criteria). These interim reports will be submitted to the NIHR (funding body for the trial), and the trial oversight committees where relevant.

**Table 2:** Progression criteria for recruitment and retention interim assessments

Progression guidance	Recruitment at 12 m	Retention at 18 m
Continue: no action needed	100%	100%
Continue: action needed	80–99%	80–99%
Continue: recovery strategy	50–79%	50–79%
Stop trial	< 50%	< 50%

#### Integrated qualitative research

In the Hand-1 feasibility study, qualitative interviews undertaken up to 8 months post-treatment demonstrated patient satisfaction with both NF and LF. However, NF has higher risks of recurrence and need for further treatment than LF in the longer term, such that patient views on treatment acceptability may change over time. In Hand-2, up to 30 semi-structured interviews will therefore be conducted with trial participants to explore their experiences and acceptability of treatment around two years after surgery, with the final sample size being determined by data saturation. Most interviews are likely to occur remotely, or face to face if preferred. Participants will be purposefully selected to ensure maximum variation in terms of age, gender, type of surgery, study centre and timing of surgery. Topic guides will be used to ensure similar topics are covered in each interview but applied in a flexible manner to enable issues of importance to emerge. The guide will focus on their experiences of living with DC pre- and post-treatment, expectations and experiences of treatment, recovery, recurrence of contractures and any additional treatment received.

### Adverse events

Both interventions within Hand-2 are minor surgical procedures that are widely available as standard care for DC within the NHS. Adverse events that could be due to the surgical procedures will be recorded as safety outcomes for the study rather than reported as adverse events. This safety data will be collected at the 6-week follow-up appointment. Serious adverse events that occur between time of surgery and the 6-week follow-up will be reported by site staff via an SAE report form emailed to the Nottingham Clinical Trials Unit (NCTU). This form will be sent to the relevant medical monitor for assessment.

### Data collection and management

Data collection will be conducted at sites and remotely, in patient’s homes. Trained site staff will carry out data collection and clinical assessments at baseline, day of treatment (or at a pre-operative appointment no more than 8 weeks before the day of treatment, if applicable), and at the 6-week follow-up timepoint which will be conducted at the patient’s standard post-operative appointment.

All other post-treatment follow-ups will be completed remotely by sending questionnaires direct to patients via email or post at 2, 3, and 4 weeks and at 3, 6, 12 and 24 months.

If remote follow-up questionnaires are not returned, the NCTU will send out a reminder letter or make a telephone call to follow up with the participant. Participants who do not receive their allocated procedure will continue to be followed up unless they opt to withdraw from the trial completely.

The Trial Management Group will monitor data collection and retention rates throughout the study.

### Data management and monitoring

Data management will be conducted by the NCTU, who will ensure the study is conducted according to Good Clinical Practice guidelines and local standard operating procedures.

All trial data will be entered on a trial-specific database with participants identified only by their unique trial number and initials. The database will be developed and maintained by NCTU. Access to the database will be restricted and secure. Data collected at sites will be either entered directly onto the database or recorded in paper worksheets and later entered onto the database. Data collected remotely will either be entered directly onto the database, if participants complete their follow-ups via a survey link sent via email, or will be entered onto paper worksheets and posted back to the NCTU if participants complete their follow-ups via post. For postal questionnaires, NCTU will complete data entry into the database.

Database validation checks, including missing data, illogical entries, values outside of expected ranges and invalid responses, will ensure data quality. Additionally, NCTU will monitor data entered by sites and raise data queries when necessary. Monitoring of study data will be completed centrally, unless triggered on-site monitoring visits are required due to persistent issues that cannot easily be rectified remotely. The chief investigator has overall responsibility for the study and is the custodian of the data.

### Statistical analyses

The analysis and reporting of the trial will be in accordance with the Consolidated Standards of Reporting Trials (CONSORT) guidelines extension for reporting non-inferiority and equivalence trials. A full statistical analysis plan will be developed and agreed prior to database lock.

A CONSORT flow diagram showing the numbers of patients approached at site, eligible, consented and randomised will be produced, and will include reasons for exclusions.

Appropriate descriptive statistics (mean, standard deviation, median, lower and upper quartiles, minimum, maximum or frequencies and percentages) for the demographic and clinical outcome measures at baseline will be used to assess balance between the randomised arms at baseline, but no formal statistical comparisons will be performed. Baseline characteristics will also be descriptively compared between those randomised and those analysed to see if the attrition has introduced any imbalances. Descriptive statistics appropriate for the outcome will also be presented for all outcomes at all collected time points by the treatment arm.

Intention to treat (ITT), analysing participants in the groups to which they were randomised, and a per-protocol analysis, excluding participants who fail to adequately adhere to the assigned treatment, will be performed for the primary outcome between group comparisons as a protection against possible ITT’s increased risk of type I error. The primary conclusion will be based on ITT, with per-protocol results used to check the consistency. For the primary outcome, a two sides 95% confidence interval (equivalently one sided 97.5% interval) for the difference in mean PEM score at 12 months between the NF and LF arms will be constructed using a linear mixed model adjusted for baseline PEM and minimisation variables. Non-inferiority of the NF will be inferred if the upper bound of this interval lies within the non-inferiority margin of 6.0 points. The mixed effects model will use all available follow-up outcome data and include a treatment-by-time interaction to estimate the between-group difference at each follow-up time point with 12 months being the primary treatment comparison.

Sensitivity analyses for the primary outcome will include:

- Complete case analysis based on observed outcome data
- Use of multiple imputation (if necessary) with auxiliary variables not included in the primary analysis also included in the imputation model
- Adjustment for the PEM score pre-intervention (in the 8 weeks before the intervention)/ day of intervention rather than the baseline PEM to explore the effect of any significant delays between randomisation and the intervention.
- Adjustment for any other baseline variable (if applicable) with a marked imbalance between the two treatment groups.

Between groups comparison of secondary outcomes will be analysed using an appropriate mixed effect model for the outcome adjusting for the same variable as the primary analysis. Complications and adverse events will be presented descriptively.

#### Procedures for missing data

We anticipate missing baseline data to be very minimal. For questionnaire outcome measures where there are published methods for dealing with missing items, these will be applied. For baseline scores which will be adjusted for as covariates, any missing data will be imputed using the mean score at each centre.

Measures will be taken to minimise missing outcome data; however, it is likely that there will be some missing data in outcome measures as participants are lost to follow-up. For the primary outcome, two principled maximum likelihood-based methods will be employed to deal with missing data, both assuming that the probability that a response is missing depends on the observed data, but not on the unobserved data i.e. the missing data is missing at random (MAR).

1. Mixed effect model as the primary analysis
2. Multiple imputation as a sensitivity analysis should there be auxiliary variables not included in the primary model: Multivariate imputation by chained equations (MICE) will be used to generate at least 20 multiply imputed datasets of each missing outcome, with an imputation model including the outcome, all predictors and other auxiliary variables to make the MAR assumption more plausible.

### Qualitative data analysis

Interviews and recruitment consultations will be audio-recorded, with permission, and transcribed verbatim in full or in parts. Interviews and recruitment consultations, along with screening logs and study documentation, will be subject to simple counts, content and thematic analyses. Findings will be compared with those from the feasibility study.

### Health economic analysis

The primary economic analysis will estimate the incremental NHS and personal social services cost per quality-adjusted life year (QALY) gained of NF versus LF at 24 months using an intention-to-treat approach. This time point is relevant for policy makers as it includes the costs and consequences of early recurrence and reoperation. The analysis will explore whether the lower initial costs of NF are subsequently offset by higher costs of recurrence and poorer patient outcomes.

The EQ-5D-5L will be valued using NICE-recommended tariffs at the time of analysis and combined with survival data to estimate QALYs. Wherever available, national unit costs (e.g. National schedule of NHS costs [29], PSSRU unit costs of health and social care [30]) will be used to value resource use. For the activity-based costing of LF and NF procedures hospital procurement costs for equipment, consumables and salaries will be utilised. Costs and outcomes beyond 12 months will be discounted at standard rates. The incremental net monetary benefit statistic, at standard NICE willingness to pay thresholds (i.e. £20,000 and £30,000 per QALY) and 95% confidence intervals will be used to summarise cost-effectiveness. The economic analysis will take an ITT approach and the prevalence of missing cost and EQ-5D-5L data will be described and multiple imputation techniques will be used as appropriate. Uncertainty will be further summarised using cost-effectiveness acceptability curves. A secondary analysis will estimate the incremental cost per difference in the primary outcome (PEM score) over the 24-month follow-up. Further secondary analyses will explore the impact of care pathways on patient costs and productivity losses. In sensitivity analyses, the robustness of our conclusions to plausible differences in key costing assumptions (e.g. the unit costs of NF and LF) will be explored. A health economic analysis plan, reviewed by the TSC, will be developed to pre-specify the methods in detail.

### Trial management and oversight

NCTU will act as co-ordinating centre for Hand-2 and will be responsible for trial management, communication with sites, and data management and storage. The Trial Management Group will be responsible for the general management of the trial and will meet monthly. Independent trial oversight will be provided by a Trial Steering Committee and a Data Monitoring Committee, who will meet at least annually to review trial progress and data. The Data Monitoring Committee is comprised of 3 independent members, whereas the Trial Steering Committee is comprised of both independent and non-independent members 84% of which are independent.

### Protocol amendments

The methods outlined in this protocol reflect the current study protocol (v3.1, 02nd November 2023). A summary of protocol amendments can be seen in the Appendix. Future amendments will follow standard notification procedures for the research ethics committee, Health Research Authority, and site investigators. Trial registries and this protocol will be issued with updates for substantial amendments.

### Confidentiality

Personal data recorded on all documents will be regarded as strictly confidential and will be handled and stored in accordance with the Data Protection Act 2018. Participants will always be identified using only their unique trial identification number, date of birth and initials.

The Coordinating Centre will maintain the confidentiality of all participant’s data and will only disclose information of participants that have given consent to any third party*.* Representatives of the Coordinating Centre and Sponsor may be required to have access to participant’s notes for quality assurance purposes but participants should be reassured that their confidentiality will be respected at all times.

### Post-trial care

Participants will continue to receive routine NHS care as appropriate upon completion of the study.

### Dissemination

The trial results will be reported in a peer-reviewed journal and presented at scientific meetings. Reporting will be in compliance with CONSORT recommendations. Results will be made available to participants if they provide consent to receive this.

## Discussion

The overall situation, with regard to the evidence base for the treatment of DC, has changed little from when the HAND-1 study was published [28, 31]. Therefore, the content of that discussion remains relevant and is significantly reproduced here.

The current lack of robust evidence on treatment for Dupuytren’s contractures of the fingers means that the choice of treatment mainly depends on surgeon and patient preferences. A comparison of NF with LF has been identified as an important research question for both surgeons and patients [14].

From a patient’s perspective, the options of NF and LF appear to offer very different short- and long-term benefits, and thus many may find one treatment option suits their lifestyle better than the other. Social circumstances, such as self-employment, duties as a carer for a relative and the financial burden of prolonged sick leave, may all influence each patient’s treatment preference, as may the desire for a straight, aesthetically satisfying finger, or to minimise the risk of needing further surgery in the future. Whilst these factors are all relevant, the evidence base against which they must be weighed is currently sub-optimal. The Hand-2 study, together with the integrated qualitative component, provides fundamental insights into the acceptability of each treatment.

The assessment of outcome of Dupuytren’s treatment with PROMs is in its infancy, and success or failure of the treatment has previously been determined in most studies by the amount of angular correction (straightening) of the flexed finger and the subsequent amount of recurrent angular deformity occurring over a pre-set time period, regardless of whether this results in the patient wishing to undergo further treatment to straighten the finger again [5, 6, 13, 32–40]. This is particularly unsatisfactory as the relationship between hand and finger function and joint-angle deformity is controversial. The HAND-2 study will provide data essential to understanding the effectiveness of LF and NF in terms of patient-reported hand function.

This study will provide much-needed robust evidence to guide clinical decision-making and inform the development of NHS guidance with regards to the surgical treatment of Dupuytren’s contractures. It will answer some of the many uncertainties regarding optimising the treatment of Dupuytren’s contractures and demonstrate the relative values of LF and NF in terms of clinical outcome, costs and acceptability to patients over a 2-year follow-up period.

### Trial status

Hand-2 is currently recruiting. The first participant was recruited in March 2022 and recruitment is expected to continue until March 2024. The trial has passed its internal pilot phase.

This publication is based upon the current version of the protocol: version 3.1 02nd November 2023.

### Supplementary Information

**Additional file 1.** Trial informed consent form (ICF).

## Data Availability

Data sharing is not applicable to this article as no datasets were generated or analysed for this protocol. Anonymised participant data generated during and analysed at the end of this trial will be made available, upon request, in accordance with the NCTU standard operating procedure after publication of trial results.

## Appendix

**Table 3:** Summary of amendments to the Hand-2 study protocol

Protocol version	Protocol date	Summary of changes
Protocol amendments prior to recruitment		
1.2	11 Nov 2021	1) Inclusion criteria updated — one or more fingers with a Dupuytren’s contracture of ≥ 30° with functional problems
		2) Sect. 8.2 — ‘2PD sensation’ removed as this measurement is not required
		3) Addition of pre-intervention assessment time point (up to
		4 weeks prior to or on the day of surgery) to allow exploring the effect of any significant delays between randomisation and intervention (due to pandemic-related increases in waiting list times)
		4) Sect. 13.4 (Procedures for missing data) under ‘missing baseline data’ to include an additional statement clarifying that where there are published methods for dealing with missing items in questionnaire outcome measures, these will be applied
		5) Needle Aponeurotomy/Needle Fasciotomy Protocol added in the appendix. This document will be used to aid Health Care Professionals 6)Statistical Methods and Sect. 13.3 CACE analysis replaced with per protocol analysis
Protocol amendments after the start of recruitment		
2.0	24 Aug 2022	1) Addition of recruitment patient pathway in Sect. 7.1.2 2) Sect. 8.2- Allows treatment to be performed in an independent sector 3) Change to the time point of pre-operation clinics assessment up to 8 weeks prior to intervention/ surgery
2.1	13 Dec 2022	1) Study duration corrected from 24 to 60 months 2) Update to co-ordinating centre contact details
2.2	12 Apr 2023	Removal of fax number for SAE reporting
3.1	02 Nov 2023	1) Correction to date of amendment for NSA01 and addition of NSA02-NSA04 to protocol amendments table
		2) Split eligibility criteria 4 into two sections for clarity
		3) Clarification that part 3 of the PEM questionnaire (Overall Assessment) forms a secondary outcome (Overall Satisfaction)
		4) Update to trial flow diagram to better reflect the patient pathway with regards to audio recording of consultations
		5) Sect. 8.1. correction to Table 1 to show that Study Finger Extension and Flexion should be measured at intervention or during 8 weeks before the intervention, in line with the schedule of assessments in Sect. 8.2
		6) Sect. 8.3. Removal of brand name of grip strength device to allow sites to use any standardised grip strength meter
		7) Sect. 9.2. Change to reportable SAEs
		8) Sect. 9.3.2. Clarification of the conditions in which the medical monitor/CI may halt the trial
		9) Sect. 1. Trial Summary Table. Correct participant population and key eligibility criteria to mention recruitment pathway 2 (hand surgery waiting lists)

## Abbreviations

DC: Dupuytren’s contractures
LF: Limited fasciectomy
NF: Needle fasciotomy
CI: Collagenase injection
RCT: Randomised control trial
PROM: Patient-reported outcome measure
PEM: Patient Evaluation Measure
SANE: Single Assessment Numeric Evaluation
EQ-5D-5L: EuroQol-5 Dimension
MYMOP: Measure Yourself Medical Outcome Profile
QRI: QuinteT Recruitment Intervention
MCP: Metacarpophalangeal
PIP: Proximal interphalangeal
DIP: Distal interphalangeal
NCTU: Nottingham Clinical Trials Unit
CONSORT: Consolidated Standards of Reporting Trials
ITT: Intention to treat
QALY: Quality-adjusted life year
