# Supplementary material for: Needle fasciotomy versus limited fasciectomy for the treatment of Dupuytren’s contractures of the fingers (Hand-2): study protocol for a randomised controlled trial
Source: Trials. 2024 Jun 19;25:398. doi: 10.1186/s13063-024-08003-1 (PMC11186219; doi:10.1186/s13063-024-08003-1)
Supplement: Supplementary file 1 — Additional file 1. Trial informed consent form (ICF). [file 13063_2024_8003_MOESM1_ESM.docx]

<To be printed on Trust headed paper>

**The Hand-2 Study**

**Informed Consent Form**

**Version 2.0 24 June 2022**

**Name of Principal Investigator**:

**IRAS Project ID: 282087**

**Participant Trial ID:**

(To be completed after randomisation)

|  | | **Please initial box** |
| --- | --- | --- |
|  | I confirm that I have read and understand the Participant Information Sheet, Version <insert current PIS version number and date > for the above study. I have had the opportunity to consider the information, ask questions and have had these answered satisfactorily. |  |
|  | I understand that my participation is voluntary and that I am free to withdraw at any time, without giving any reason, and without my medical care or legal rights being affected. I understand that should I withdraw, then the information collected so far cannot be deleted and that this information may still be used in the study analysis. |  |
|  | I understand that relevant sections of my medical notes and data collected in the study may be looked at by authorised individuals from the Nottingham Clinical Trials Unit (University of Nottingham), the Sponsor (Nottingham university Hospitals NHS Trust) NHS bodies, the study research group and regulatory authorities where it is relevant to my taking part in this study. I give permission for these individuals to have access to these records and for a copy of this signed consent form to be sent to the Nottingham Clinical Trials Unit. |  |
|  | I give permission for the Nottingham Clinical Trials Unit, the Sponsor and the study research group to collect, store, analyse and publish information obtained from my participation in this trial. I understand that my personal details will be kept confidential. |  |
|  | I understand that the Nottingham Clinical Trials Unit and the study research group will be provided with my personal details (phone number and email address (postal address optional) to contact me for the purpose of obtaining information and sending study communications for up to 5 years after my treatment. I give my permission for this information to be kept and for these individuals to contact me. |  |
|  | I agree to my contact details being sent to a researcher at the University of Bristol so I can be contacted regarding having a discussion with them about my experiences of the treatment I received in the study |  |
|  | I understand that the information held and maintained by my GP, NHS Digital and other central UK NHS bodies may be used to help contact me or provide information about my health status |  |
|  | I agree to my GP being informed of my participation in this study. |  |
|  | I understand that the anonymised information collected about me may be used to support other research in the future and may be shared with other researchers. |  |
|  | I agree to take part in the above study. |  |

|  | | **Please initial either box** | |
| --- | --- | --- | --- |
|  | **Optional** | **Yes** | **No** |
|  | Once the study has ended I would like to know what the study found out and agree for my email address ,or address to be collected and used for sending me this |  |  |
|  | I agree to be contacted and informed about future studies. I understand that there is no obligation and I will just be informed of what the future study will involve. |  |  |
|  | I understand that my name and telephone number will be held by Esendex (text messaging provider) and their sub-processors and will be used to contact me by text message. I give permission for this information to be retained by Esendex for two years or until the end of the study (whichever occurs first). I understand that if I withdraw my personal details will be deleted. |  |  |

_______________________ ______________ _______________________________

Name of Participant Date Signature

_______________________ ______________ _______________________________

Name of person taking consent Date Signature

(You must be on the delegation log)

*Original signed ICF to be kept in the Investigator Site File. 3 copies: 1 for participant, 1 for the medical notes and 1 to be sent to the Nottingham Clinical Trials Unit.*
